# DeepSA: a deep-learning driven predictor of compound synthesis accessibility

**DOI:** 10.1186/s13321-023-00771-3

**Published:** 2023-11-02

**Authors:** Shihang Wang, Lin Wang, Fenglei Li, Fang Bai

**Affiliations:** 1 Shanghai Institute for Advanced Immunochemical Studies and School of Life Science and Technology, ShanghaiTech University, 393 Middle Huaxia Road, Shanghai, 201210 China; 2 School of Information Science and Technology, ShanghaiTech University, 393 Middle Huaxia Road, Shanghai, 201210 China; 3 Shanghai Clinical Research and Trial Center, Shanghai, 201210 China

**Keywords:** Synthetic accessibility, Drug design, Deep learning, Chemical language model

## Abstract

With the continuous development of artificial intelligence technology, more and more computational models for generating new molecules are being developed. However, we are often confronted with the question of whether these compounds are easy or difficult to synthesize, which refers to synthetic accessibility of compounds. In this study, a deep learning based computational model called DeepSA, was proposed to predict the synthesis accessibility of compounds, which provides a useful tool to choose molecules. DeepSA is a chemical language model that was developed by training on a dataset of 3,593,053 molecules using various natural language processing (NLP) algorithms, offering advantages over state-of-the-art methods and having a much higher area under the receiver operating characteristic curve (AUROC), i.e., 89.6%, in discriminating those molecules that are difficult to synthesize. This helps users select less expensive molecules for synthesis, reducing the time and cost required for drug discovery and development. Interestingly, a comparison of DeepSA with a Graph Attention-based method shows that using SMILES alone can also efficiently visualize and extract compound’s informative features. DeepSA is available online on the below web server (https://bailab.siais.shanghaitech.edu.cn/services/deepsa/) of our group, and the code is available at https://github.com/Shihang-Wang-58/DeepSA.

**Supplementary Information:**

The online version contains supplementary material available at 10.1186/s13321-023-00771-3.

## Introduction

Computer aided drug design (CADD), especially AI aided drug design (AIDD), has become an important tool in modern innovative drug discovery and development, which can significantly expediate the drug development process and cut investment costs [1–4]. Fragment-based drug design (FBDD) is a classical CADD strategy. Based on a target structure, FBDD performs virtual screening (VS) from the molecular fragment library to obtain ligand fragments, and many optimization and transformation steps are carried out according to the structural information of the target protein to obtain new compounds with high affinity and achieve the goal of lead compound design [5–7]. In this situation, the designed molecules often get stuck with difficulty in synthesizability.

In addition, with the continuous development of artificial intelligence technology, an increasing number of computational molecular generation models are developed based on various of artificial intelligence algorithms, including variational auto encoders (VAE) [8], generation of confrontation networks (GAN) [9], reinforcement learning (RL) [10–12], flow-based generation models and diffusion models [13, 14]. These models can generate new hit compounds for the known targets of existing diseases and optimize the structure of existing lead compounds. They help medicinal chemists to find useful molecules from the vast chemical space and shorten the time for drug discovery and development [15, 16]. However, most new molecules created by generation models often face major challenges in terms of synthetic accessibility [17].

The synthesizability prediction can be seen as a large data-required complicated problem, and machine learning is suitable for dealing with such problems. A huge training dataset of molecules and their pre-defined “synthesizability scores” is the prerequisite. Then an artificial intelligence model is able to be designed to learn the relationship between the molecule structures and their synthesizability. So far, several such tools have been developed, such as SAscore (Synthetic Accessibility score), which assesses the compositional fragments and complexity of molecules by analyzing the historical synthesis knowledge obtained from the information of millions of synthesized chemicals, and finally outputs a score in the range of 1 to 10 [18]. This method performs better than the other methods, including SCScore (Synthetic Complexity score) [19], RAscore (Retrosynthetic Accessibility score) [20] and SYBA (SYnthetic Bayesian Accessibility) [21], from an evaluation study from Skoraczyński et al. [22]. SCScore is a method for quantifying synthesis complexity, which uses deep neural networks and trains on a set of 12 million reactions obtained from the Reaxys database and the output score for evaluation ranges from 1 to 5 [19]. RAscore is a machine learning classifier trained with more than 300,000 compounds from the ChEMBL database [20]. SYBA uses Bernoulli Naive Bayes classifier to evaluate whether a given molecule is easy- (ES) or hard-to-synthesize (HS) [21]. Unlike the other mentioned methods, SYBA assesses each fragment of a molecule with an assigned SYBA score to label its synthesizability. RetroGNN is a machine learning-driven method to estimate synthesizability via approximating the outputs of a retrosynthesis planning software within a given search space [23]. The latest synthetic accessibility evaluation model is GASA (Graph Attention-based assessment of Synthetic Accessibility), which is a graph-based method for predicting the synthetic accessibility. Small organic compounds are classified as ES or HS based on the capturing of the local atomic environment by leveraging information from neighboring nodes through attention mechanisms and enrichment of the overall training process by incorporating bond features to obtain a more complete understanding of the global molecular structure [24]. GASA has been reported as one of the state-of-the-art models, which has shown remarkable performance in distinguishing the synthetic accessibility of similar compounds, with strong interpretability and generalization ability, significantly outperforming other existing methods [24]. These methods were trained on diverse compound datasets and could be divided into structure-based (SAscore and SYBA) and reaction-based (SCScore, RAscore, RetroGNN and GASA) [18–24]. Interestingly, these reaction-based methods used different reaction datasets. SCScore was trained using 12 million chemical reactions from the Reaxys database, while RAscore, RetroGNN, and GASA used three different retrosynthesis analysis softwares, AiZynthFinder [25], Molecule.one (https://www.molecule.one/), and Retro* [26], respectively, to generate synthesis routes for model training.

In this study, we propose a new model for evaluating the synthetic accessibility of compounds based on the chemical language model named DeepSA. This model can differentiate easy-to-synthesize from that are hard-to-synthesize with a much higher accuracy rate. We compared the discriminative ability of DeepSA with other existing models for evaluating the synthetic accessibility of compounds (GASA, SYBA, RAscore, SCScore, and SAscore). The results show that the performance of DeepSA is particularly well as it more accurately assesses the synthetic difficulty of real drug molecules in existing research reports. We have deposited the original code of DeepSA on GitHub (https://github.com/Shihang-Wang-58/DeepSA) and also provided an online platform for the public to use DeepSA (https://bailab.siais.shanghaitech.edu.cn/services/deepsa/).

## Materials and methods

### Collection of datasets

To ensure a fair comparison with existing methods, we used the same datasets in this study as Yu et al. [24] to train the model for predicting synthesis accessibility of molecules. The datasets consist of two parts. The first part is used for training DeepSA, and the other part is used to evaluate the performance of DeepSA and other synthetic accessibility models. Among them, hard-to-synthesize molecules are marked as positive samples and easy-to-synthesize molecules are marked as negative samples.

The training dataset contains 800,000 molecules, of which 150,000 are from the ChEMBL [27] or GDBChEMBL [28], and has been labeled the synthetic accessibility by a multi-step retrosynthetic planning algorithm called Retro* [26], which is a neural-based A*-like algorithm that can efficiently find simplified synthetic routes for aimed compounds. In this study, we used the default parameters suggested from the developers of Retro* to analyze the synthesis steps. The training data for Retro* are from the USPTO reaction dataset and a list of commercially available building blocks from *eMolecules* [26]. The detailed description for the parameter settings for Retro* has been listed in Additional file 1: Table S1. Simply input the SMILES of the molecule to Retro *, and it will output the synthesis route of the molecule and the final number of synthesis steps. A molecule requires less than or equal to 10 synthetic steps was labeled as ES, otherwise, if the required step is larger than 10 or can’t be successfully predicted by Retro* was labeled as HS. Another 650,000 molecules were derived from SYBA [21], with positive samples coming from purchasable molecules in the ZINC15 database [29] and negative samples generated by the Nonpher algorithm [30]. All samples were divided into a training set, and a test set was set as 9:1 ratio. Meanwhile, we amplified different SMILES representations of the same molecule to add advanced sampling operations to the dataset.

The independent test sets used to evaluate the performance of the various models consist of three parts. In summary, the three datasets were drawn from three previous published works. Independent test set 1 (TS1) contains 3,581 ES and 3,581 HS molecules that obtained directly from the study of SYBA [21, 24]. The independent test set 2 (TS2) contains 30,348 molecules derived from the study of RAscore [20]. The independent test set 3 (TS3) consists of 900 ES and 900 HS molecules which obtained from the study of GASA [24]. The compounds in TS3 were collected from different sources and have higher similarity of fingerprints, which makes the prediction task more challenging. There was no overlap between the training set and the independent test sets. The data sets used in this study are shown in Additional file 1: Table S2. Finally, to further verify the performance of DeepSA for compounds with real synthetic pathways, we selected 18 compounds with complete synthetic pathways from the published literatures [31–48], which will be detailed introduced in the section of *Results and Discussion*.

### Criteria for Performance Evaluation

Model evaluation is an important part for classification tasks. When comparing the predictive performance of different models, most evaluation indicators can only show the predictive performance of the model from a particular aspect. Therefore, we used several statistical indicators, including accuracy (*ACC*, Eq. (1)), *Precision* (Eq. (2)), *Recall* (Eq. (3)), *F–score* (Eq. (4)) and the *AUROC* [49–52]. *ACC* indicates the prediction accuracy; however, if the sample size belonging to different classes in the data is uneven, the conclusions of the evaluation of *ACC* may be questionable. *Precision* represents the proportion of correctly predicted positive set out of all predicted positive set. *Recall* is the proportion of correctly predicted positive set out of all positive set. The *F–score* is defined as the harmonic mean of the model’s precision and recall. ROC curve is an important index for evaluating the generalization performance of models.

1 $$\begin{array}{c}ACC=\frac{TP+TN}{TP+TN+FP+FN}\end{array}$$

2 $$\begin{array}{c}Precision=\frac{TP}{TP+FP}\end{array}$$

3 $$\begin{array}{c}Recall=\frac{TP}{TP+FN}\end{array}$$

4 $$\begin{array}{c}F{-}score=\frac{2\,*\,Precision\,*\,Recall}{Precision\,+\,Recall}\end{array}$$

*TP* and *FN* indicate that if the true label of the sample is positive, the prediction labels are positive and negative, respectively. *TN* and *FP* indicate that if the true label of the sample is negative, the prediction labels are negative and positive, respectively.

### Classification threshold of the models

In this study, compared with DeepSA, GASA and RAscore are binary classification models with an output probability between 0 and 1. However, the output of SYBA, SAscore and SCscore is a non-binary score. To compare the performance of all models more fairly, two classification thresholds (cut-offs) were used to evaluate our method, one is 0.5 which is the same as the other binary-classification methods, e.g., GASA and RAscore, as reported in Yu et al. [24], and the other is 0.47, which was the optimal cut-off of ROC determined using the training data. The prediction results of each method were analyzed using scikit-learn [53] to calculate the *ACC*, *Recall*, *Precision*, and *F–score*. For DeepSA, SAscore and SCscore, output scores above the threshold are considered as HS and below the threshold are considered as ES. For GASA, RAscore and SYBA, on the contrary, output scores above the threshold are considered as ES and below the threshold are considered as HS.

### Network architecture of DeepSA

**Fig. 1:**
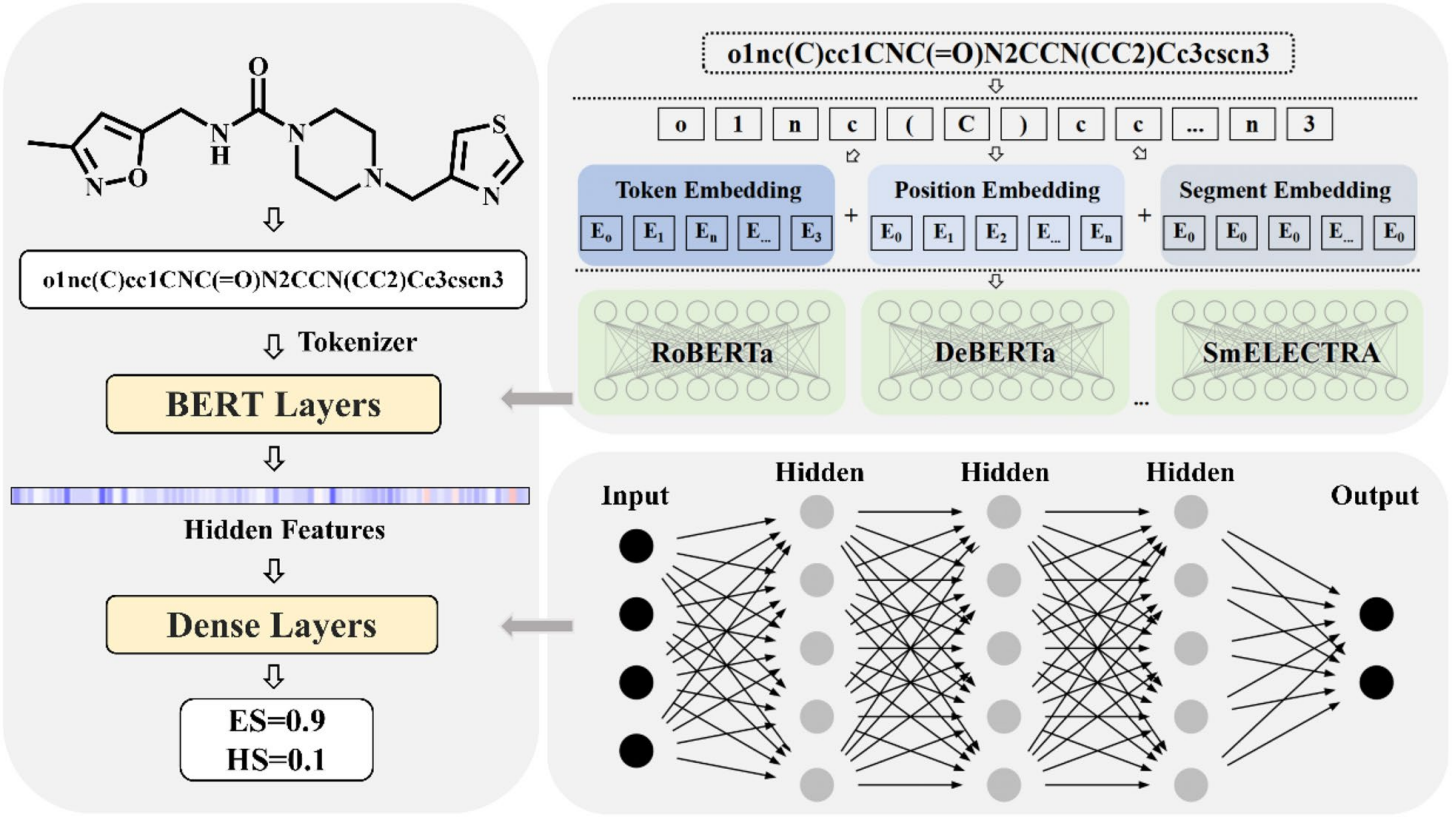
An illustration of the designed architecture for DeepSA. We designed and trained Bidirectional Encoder Representations from Transformers (BERT) model and fine-tuned on our labeled synthesis related data to evaluate synthesizability from the given molecules’ SMILES. A dense layer was added following the BERT layer to perform binary classification task. If the given score is equal to or greater than 0.5, the molecule is considered difficult to synthesize; otherwise, if the score is less than 0.5, it will be easy to obtain

The DeepSA proposed in this study consists of three modules: the data processing module, the feature embedding module, and the decoder module. The architecture of DeepSA is shown in Fig. 1. We converted the original SMILES in dataset to canonical SMILES, and then the dataset was further expanded by introducing alternative formats of SMILES of some randomly selected molecules by RDKit [54]. The final size of training, and test dataset after data enhancement are 3,593,053 and 399,216. Meanwhile, a Byte-Pair Encoder (BPE) tokenizer from HuggingFace tokenizer library was used to encode the structures for compounds, which can treat the basic atomic and ring structures in the input SMILES as “words” or “sentences” and make meaningful predictions using the previous reported ChemBERTa SMILES-Tokenizer [55, 56]. Firstly, we collected a number of network architectures from different natural language models, including bert-mini (MinBert) [57], bert-tini (TinBert) [57], roberta-base (RoBERTa) [58], deberta-v3-base (DeBERTa) [59], Chem_GraphCodeBert (GraphCodeBert) [60] and electra-small-discriminator (SmELECTRA) [61], and two chemical language models, including ChemBERTa-77M-MTR (ChemMTR) [62] and ChemBERTa-77M-MLM (ChenMLM) [62]. Secondly, we trained DeepSA models based on these different natural language models and two chemical language models using the designed synthetic accessibility dataset of molecules. The architectures of these natural language models were fine-tuned on the enhanced dataset to adopt the synthetic accessibility prediction task. The AutoGluon package [63, 64] was employed to fine-tuning stages. The learning rate was set to 0.001 and adjusted during training process by cosine decay schedule. The training process was performed for a maximum of 20 epochs, and the validated every 0.2 epoch, and up to three checkpoint models with the highest *ACC* on the validation set were stored. Finally, the top three models were fused by the greedy soup method [65] and the final DeepSA model was generated. The whole training process was performed on a RTX3090 GPU. Detailed information regarding to output dimensions of each layer and hyperparameters utilized in the DeepSA model are listed in Additional file 1: Table S3.

## Results and discussion

### Proposing the DeepSA models for predicting synthetic accessibility for molecules

In recent years, the development of natural language processing techniques has led to the emergence of numerous natural language models, providing a range of frameworks to process protein sequence data like natural languages. Intuitively, SMILES sequences used to represent compounds share certain similarities with natural language. Both are composed of a diverse vocabulary of simple characters, and generate complex sentences through simple rules. This similarity has inspired researchers to transfer the framework of natural language models to compound data, with the aim of training chemical language models, e.g., SMILES-BERT [66], in order to achieve improved performance in compound-related tasks. Therefore, we aim to explore whether a training strategy similar to text classification tasks in natural language can be employed to the chemical language models for evaluating the synthesizability of molecules. This will further enhance our understanding of chemical language models and the synthesizability of compounds.

In this study, a chemical language framework to predict the synthetic accessibility for molecules was designed, where we tried various language models as the encode layer, such as bert-mini (MinBert) [57], bert-tini (TinBert) [57], roberta-base (RoBERTa) [58], deberta-v3-base (DeBERTa) [59], Chem_GraphCodeBert (GraphCodeBert) [60], electra-small-discriminator (SmELECTRA) [61], ChemBERTa-77M-MTR (ChemMTR) [62] and ChemBERTa-77M-MLM (ChenMLM) [62]. First, molecule structures are converted into SMILES strings, which are then encoded into embeddings using a BPE tokenizer. Subsequently, the embeddings are further processed using various techniques, such as positional embeddings. Finally, through multiple encoding layers and linear layers, the model outputs probabilities for the two classification categories of easy synthesis (ES) and hard synthesis (HS) using a softmax activation function. The performance of DeepSA on the test set was shown in Table 1. It is great to see high *ACC*, *recall*, *precision*, and *F–score* on the test results. The high *precision* of the models reduces the risk of misjudging ES compounds as HS, which improves decision accuracy. Additionally, the high *recall* of the models means that they can identify more truly ES compounds, which improves decision comprehensiveness and reliability. It is also impressive that almost all of the models had *AUROC* values higher than 0.98. Next, we will further evaluate the model's generalization ability on the independent test sets.

**Table 1:** Performance comparison of the different models on the test set

Model	*ACC*	*Recall*	*Precision*	*F–score*	*AUROC*
DeepSA_ChemMTR	0.971	0.968	0.974	0.971	0.997
DeepSA_ChemMLM	0.961	0.955	0.967	0.961	0.995
DeepSA_MinBert	0.939	0.933	0.945	0.939	0.988
DeepSA_TinBert	0.942	0.937	0.947	0.942	0.990
DeepSA_RoBERTa	0.940	0.940	0.940	0.940	0.988
DeepSA_DeBERTa	0.898	0.873	0.920	0.896	0.959
DeepSA_GraphCodeBert	0.938	0.931	0.944	0.937	0.987
DeepSA_SmELECTRA	0.944	0.938	0.949	0.943	0.990

### Performance comparison of different synthetic accessibility prediction models

**Fig. 2:**
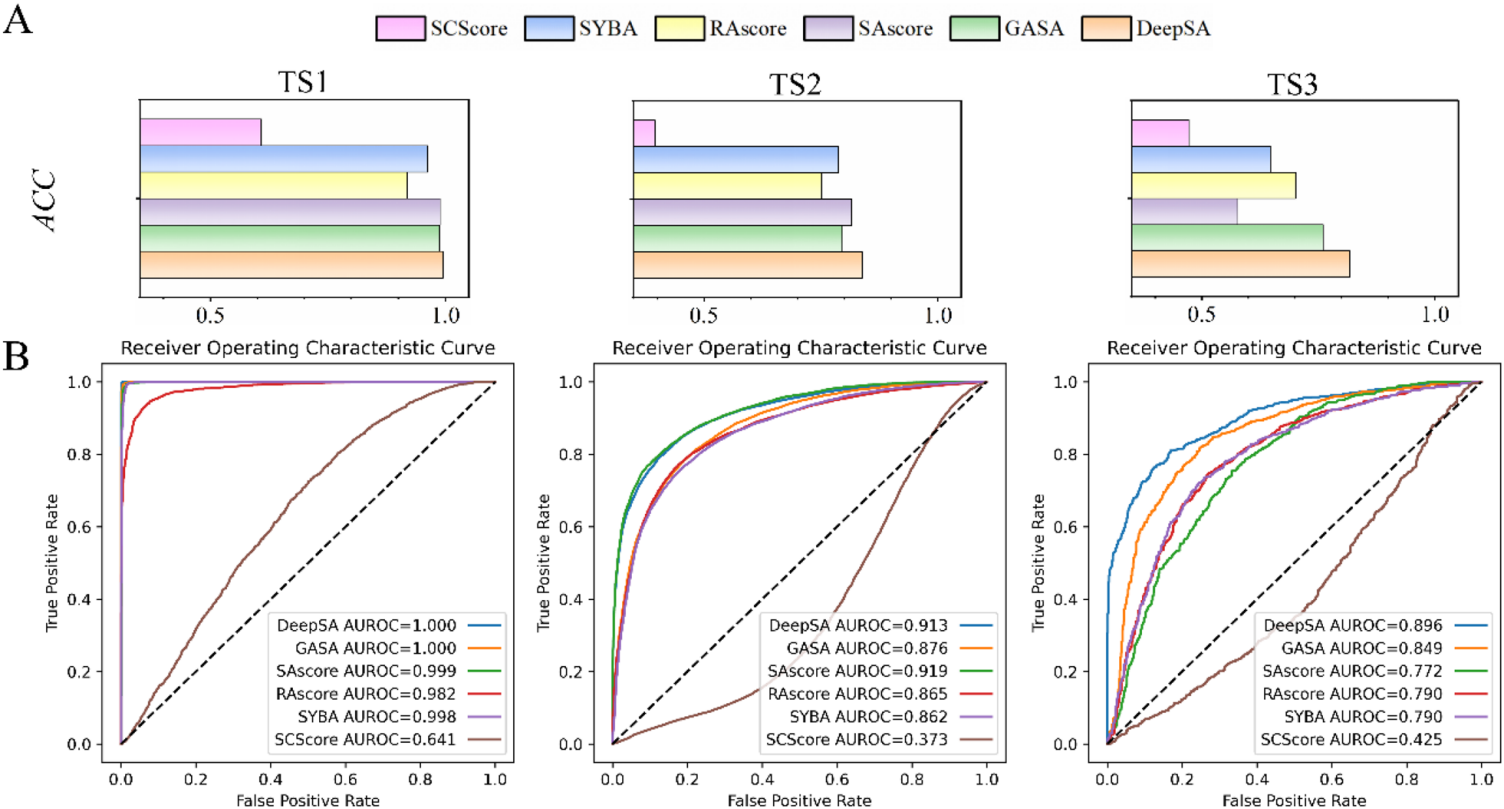
**A** *ACC* of the different synthetic accessibility classification methods over the three independent test sets. **B** ROC curves of the above methods on the three independent test sets. DeepSA shows higher early enrichment rates

Three independent test sets TS1, TS2, and TS3 are used to compare DeepSA with some state-of-the-art molecular synthesis accessibility assessment methods. The results have been summarized in Table 2, and it is shown that DeepSA can perfectly discriminate between ES and HS on TS1, only slightly lower than SAscore on TS2, and outperforms all existing methods on the most challenging TS3, indicating its excellent performance in identifying the difficulty of synthesis of similar compounds (Table 2 and Additional file 1: Table S4). DeepSA based on SmELECTRA model showed better performance on TS3, reflecting the difficulty of molecular synthesis in the real world (Fig. 2A), so we used DeepSA_SmELECTRA as the standard DeepSA model for the following analysis.

**Table 2:** Performance comparison of the different models on the external three test sets

Datasets	Model	*ACC*	*Recall*	*Precision*	*F-score*	*AUROC*	Threshold
TS1	DeepSA	0.995	1.000	0.989	0.995	1.000	0.47
	DeepSA	0.995	1.000	0.990	0.995	1.000	0.50
	GASA	0.987	0.999	0.976	0.987	1.000	0.50
	SAscore	0.989	0.992	0.986	0.989	0.999	4.50
	SAscore	0.665	0.331	0.998	0.497	0.999	6.00
	RAscore	0.919	0.867	0.967	0.914	0.982	0.50
	SYBA	0.962	1.000	0.930	0.964	0.998	0.00
	SCScore	0.608	0.698	0.592	0.641	0.641	3.10
TS2	DeepSA	0.840	0.746	0.861	0.799	0.913	0.47
	DeepSA	0.838	0.730	0.871	0.795	0.913	0.50
	GASA	0.796	0.677	0.815	0.740	0.876	0.50
	SAscore	0.815	0.603	0.946	0.737	0.919	3.40
	SAscore	0.664	0.216	0.996	0.355	0.919	6.00
	RAscore	0.751	0.485	0.878	0.625	0.865	0.50
	SYBA	0.787	0.627	0.834	0.716	0.862	0.00
	SCScore	0.395	0.442	0.341	0.385	0.373	2.30
TS3	DeepSA	0.819	0.761	0.861	0.808	0.896	0.47
	DeepSA	0.817	0.753	0.864	0.805	0.896	0.50
	GASA	0.760	0.646	0.837	0.729	0.849	0.50
	SAscore	0.577	0.211	0.788	0.333	0.772	3.10
	SAscore	0.512	0.044	0.690	0.084	0.772	6.00
	RAscore	0.701	0.571	0.772	0.656	0.790	0.50
	SYBA	0.647	0.387	0.806	0.523	0.790	0.00
	SCScore	0.472	0.723	0.481	0.578	0.425	2.20

As shown in Fig. 2B, the ROC curves for existing methods that evaluate synthetic accessibility of compounds, including GASA, SYBA, SCscore, SAscore, and RAscore. DeepSA achieves a significantly higher early enrichment rate than GASA and other models in identifying HS.

**Fig. 3:**
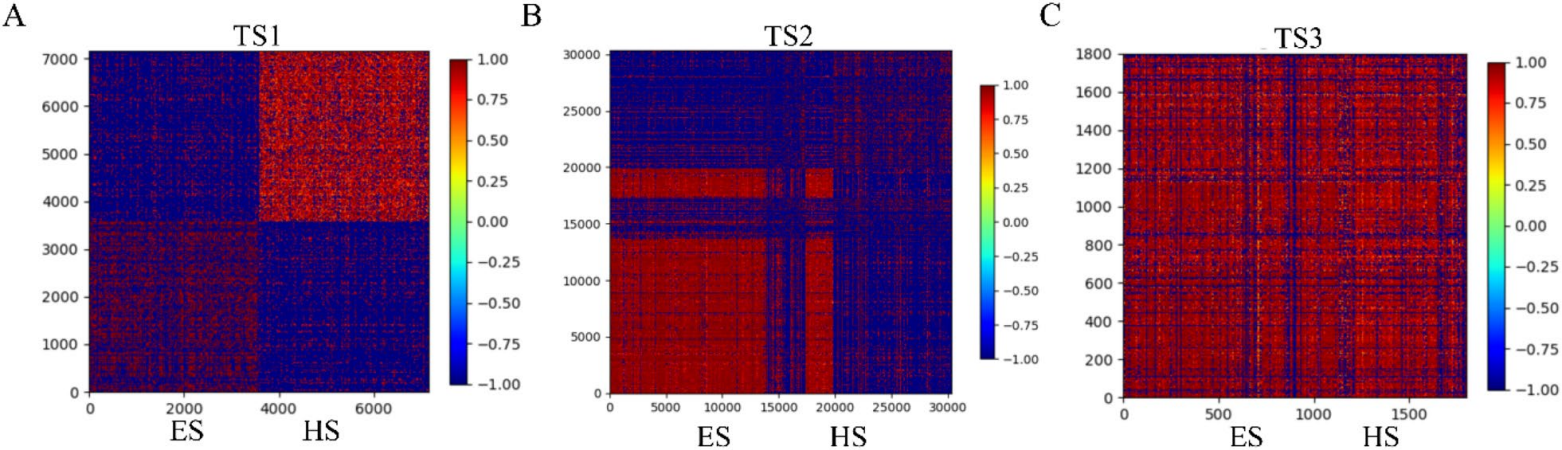
Heatmaps of fingerprint similarities between the ES and HS molecules in three independent test sets: TS1, TS2, and TS3. **A** The ES and HS compounds in TS1 have high fingerprint similarity within their own groups, but significant different patterns presenting between the groups of ES and HS. **B** Small portion of molecules of ES and HS show similar fingerprint patterns. **C** All compounds in TS3 have high fingerprint similarity, indicating that this is the most difficult test set for the classification task

As shown in Table 2 and Fig. 2, the prediction accuracy of the model decreases sequentially on TS1, TS2 and TS3, which may be due to various molecular properties among these three independent test sets. Therefore, the topological torsion fingerprint and Yule similarity metric were used to calculate the similarity matrix for these three datasets and the similarity matrix was presented as heatmap in Fig. 3. The groups of ES and HS molecules in TS1 show high fingerprint similarity within each group. Comparatively significant differences can be seen between the ES and HS groups. It indicates that the difference in patterns of the molecular embeddings may be great helpful for telling HS from ES (Fig. 3A). Figure 3B presents the fingerprint patterns for the molecules in TS2. Most of them show a clear difference between HS and ES, but a few show similar patterns between HS and ES, which presents some difficulty in prediction. Unlike TS1 and TS2, the fingerprints for HS and ES in TS3 have very similar patterns, indicating the challenges in the prediction (Fig. 3C).

**Fig. 4:**
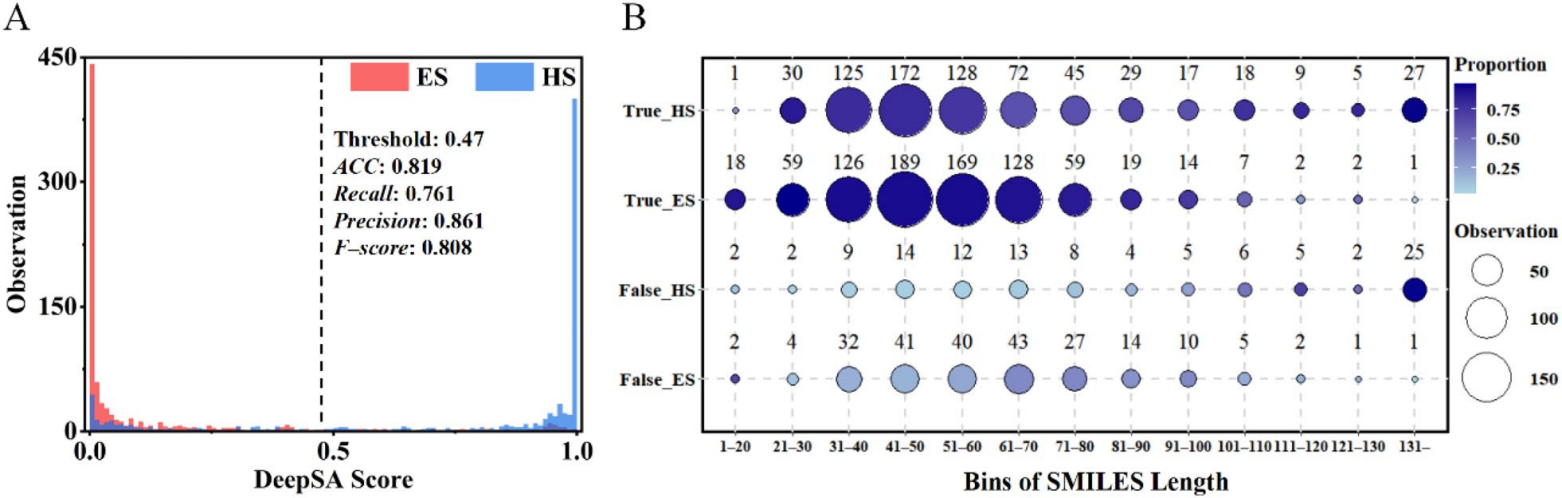
Prediction results for 1,800 compounds in TS3. **A **Score distribution histogram for the results from DeepSA over TS3. **B** The bubble plot of SMILES length versus synthetic accessibility prediction for 1800 compounds in TS3. The vertical coordinates represent the prediction outcome of the model. True_HS represents true positives, True_ES is true negatives, False_HS indicates false positives, and False_ES represents false negatives. Bubble size represents the number of observations in the SMILES length intervals. The depth of the bubble color represents the proportion of the prediction outcome in each bin of the SMILES length of compounds

TS3 is the test set created by medicinal chemists that is closer to chemical synthesis tasks. Therefore, TS3 is the one of the most suitable datasets for validating the performance of different models. Since DeepSA is a chemical language model inspired by language models in the field of NLP, intuitionally, the length of the “sentences” (SMILES) may have an impact on the performance of a language model. To answer this question, we took TS3 to investigate the relationship between the model’s performance and the length of SMILES. However, in doing so, we found that there doesn’t appear to be a strong correlation between synthetic complexity and the length of the compounds’ SMILES (Fig. 4A). Although the percentage of False_HS increases somewhat in the long SMILES region (Fig. 4B); we still can’t determine whether the performance of our model is sensitive to the length of SMILE based on the results over such a limited database. Overall, DeepSA passes the test when predicting non-extreme molecules. As the TS3 ROC curves shown in Fig. 2B, DeepSA has a much higher early enrichment rate on discriminating the molecules which are difficult to be synthesized compared to other models, thus helping users to avoid the highly cost molecules for synthesis, thereby saving the resource and reducing the time for the experimental validation of compounds designed from molecule generation model.

### Generalization ability and robustness of DeepSA model

To further verify the predictive performance of DeepSA for compounds with real synthetic pathways, we tested 18 compounds which have published synthetic pathways (those are real synthesis pathways instead of retrosynthetic analysis or chemist created) outside of the training set and the independent test set (Table 3), and predicted synthetic accessibility scores for these compounds using DeepSA and other synthetic accessibility assessment methods. The results showed that DeepSA successfully distinguished the synthetic difficulty labels of all compounds when divided by 10 synthetic steps.

**Table 3:** Prediction results for the different types of molecules

Compound name	2D	DeepSA	GASA	SAscore	RAscore	SYBA	SCScore	Synthesis steps	Refs.
Cularine		0.999	0.458	2.933	0.939	−0.556	3.706	16	[31]
Goniomitine		0.999	0.165	3.831	0.314	−16.213	4.376	12	[32]
Fusaequisin A		0.976	0.008	5.066	0.023	−51.766	3.925	20	[33]
Haliclonin A		0.960	0.002	6.513	0.131	−97.488	3.982	38	[34]
Hyacinthacine A1		0.959	0.108	3.847	0.638	−17.617	3.446	13	[35]
Hydroxyancepsenolide		0.744	0.029	3.892	0.018	−33.231	4.000	13	[36]
Kirkamide		0.630	0.475	4.142	0.264	−16.188	3.161	11	[37]
Longianone		0.727	0.190	4.666	0.668	−45.744	2.857	14	[38]
Simpotentin		0.522	0.075	4.560	0.201	25.489	3.643	14	[39]
Halomon		0.403	0.688	4.847	0.302	−6.849	2.588	10	[40]
Dihydropinidine		0.340	0.455	3.275	0.927	−29.819	3.102	9	[41]
Scorodonin		0.306	0.452	4.831	0.907	−26.430	2.160	6	[42]
Pinnatolide		0.294	0.718	3.534	0.547	−6.103	2.498	7	[43]
Tanikolide		0.289	0.371	3.072	0.797	2.726	3.218	7	[44]
Sedridine		0.128	0.924	3.324	0.990	−23.154	2.987	7	[45]
Isolaurepan		0.117	0.958	2.790	0.393	22.754	3.609	4	[46]
cis-Perhydroazulene		0.086	0.724	2.532	0.965	10.031	2.746	8	[47]
Gabapentin		0.071	0.770	2.400	0.973	2.213	2.161	3	[48]

**Fig. 5:**
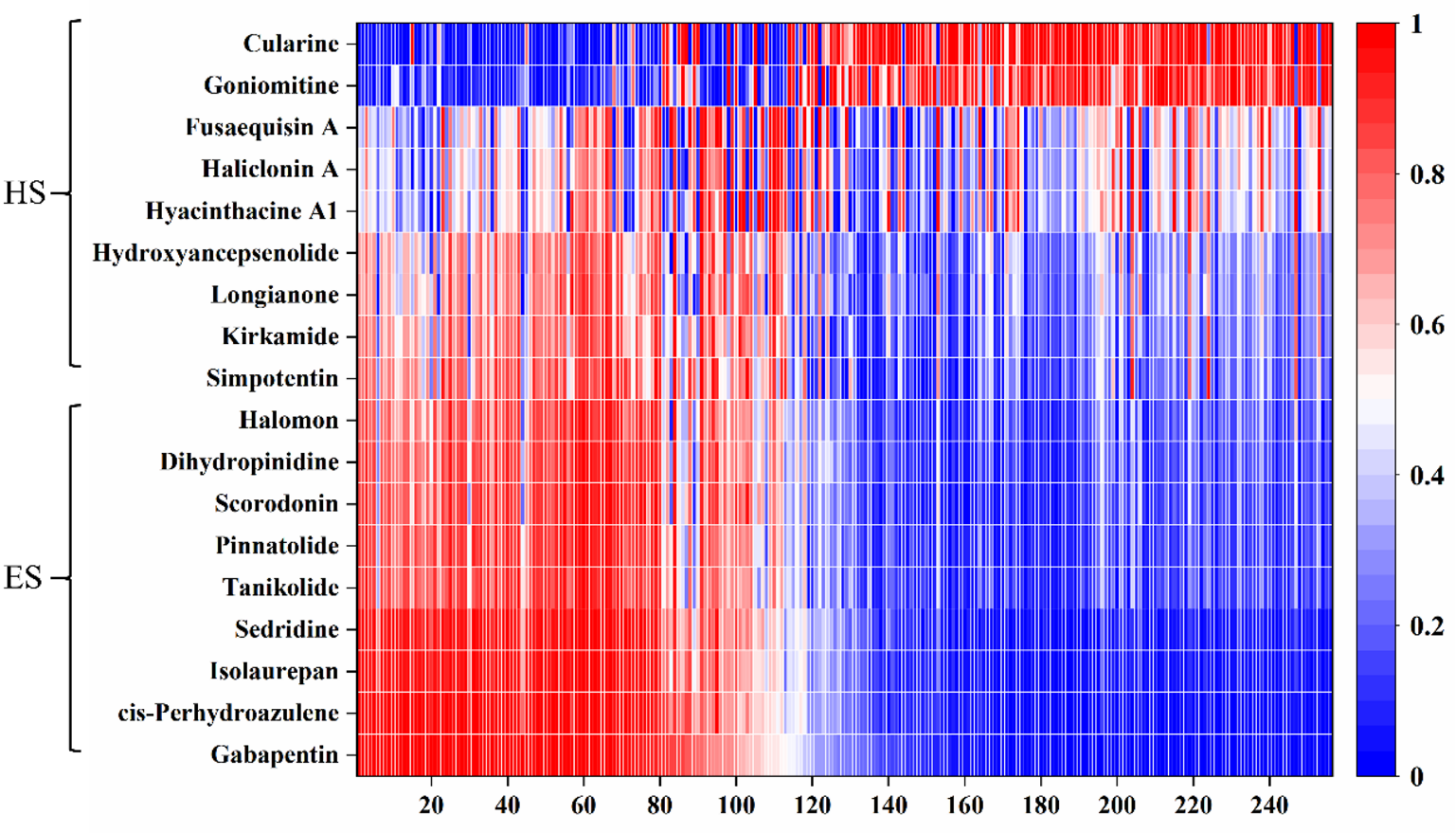
Embeddings of 18 different selected compounds in DeepSA. Each compound was represented as a matrix of 256 dimensional vectors. The matrix is normalized by column and uniformly ordered

Also, we checked the embedding of these compounds in DeepSA, where each compound is represented as a matrix of 256-dimensional vectors. Since DeepSA is a chemical language model, we explored whether difference in randomized SMILES of the same compound make any effects on the embedding. We generated three different randomized SMILES representations for each compound and extracted a total of 54 embeddings of the 18 compounds described above (Additional file 1: Table S5). Ultimately, we normalized all the embeddings and used the heatmap (Fig. 5 and Additional file 2: Fig. S1) to visualize their embedding patterns. We found that the embeddings between HS and ES are significantly different in most regions along the dimension vectors. The vector size of the first hundred dimensions of ES is obviously higher than that of HS, while the opposite is true for the last hundred dimensions. The pattern differences within the group of HS or ES are mainly in the middle range of the dimension vectors. Meanwhile, differences in the embeddings correspond to the predicted scores for molecules’ synthetic accessibility. If a compound is predicted to have a higher probability of being HS or ES, it will be more clearly marked in the embeddings. For the same compound represented by different randomized SMILES, the embeddings mostly showed a high degree of similarity, which might indicate that different randomized SMILES of the same compound don’t affect the prediction accuracy of synthetic accessibility, showing that the DeepSA model has a certain robustness.

### Constructing web server for public use

**Fig. 6:**
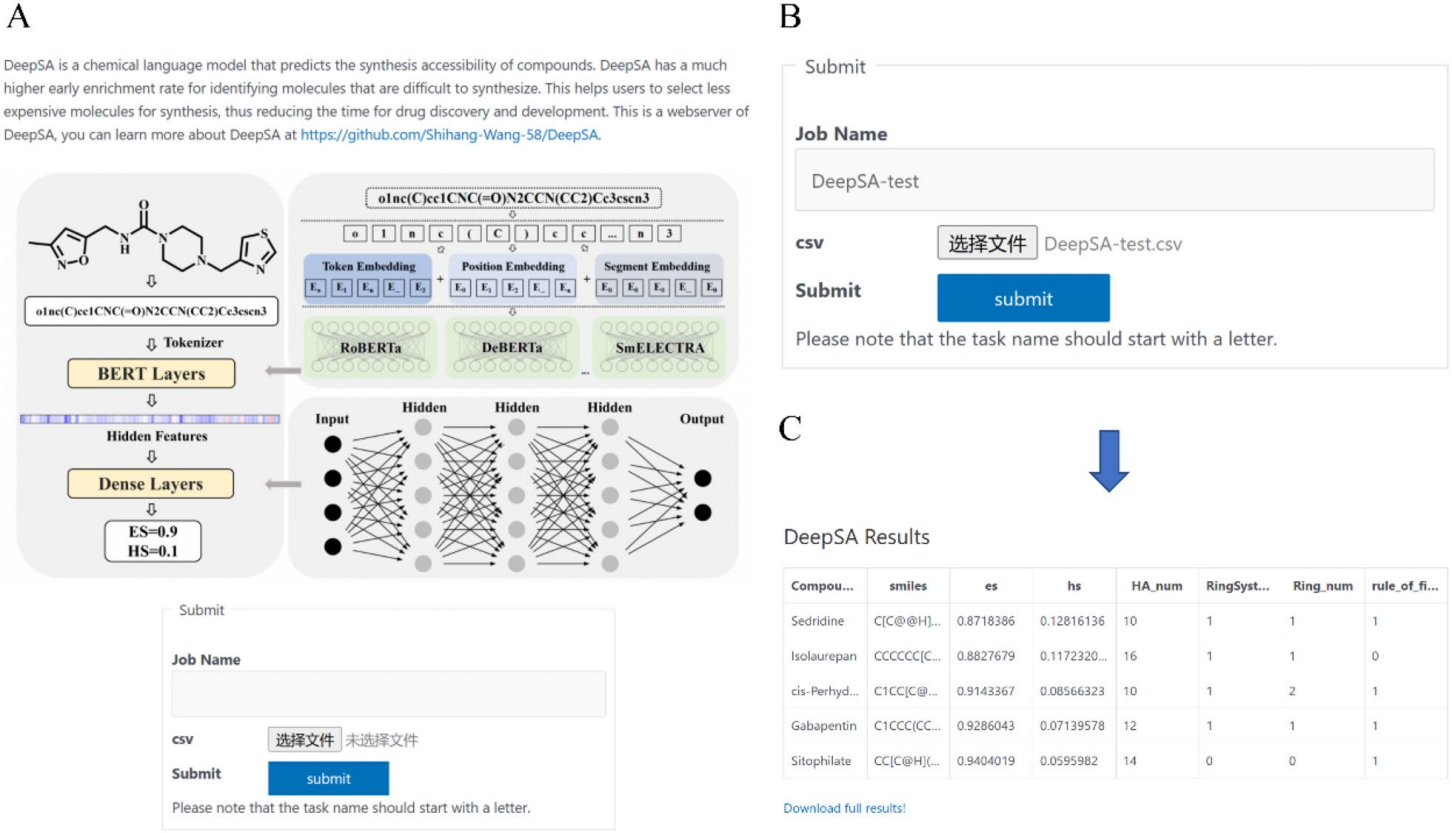
Screenshots of DeepSA web server. **A** The web interface; **B** Input data upload and information filling interface; **C** The rsults download page contains the synthetic accessibility prediction scores for submitted molecules, as well as the structural information of these molecules for the correctness check of the input

To facilitate the use of our model by biomedical researchers, a trained model was deployed on a web server that can be publicly accessed by https://bailab.siais.shanghaitech.edu.cn/services/deepsa/ (Fig. 6A). Users can upload a molecule file in csv format which contains the SMILES of molecules desired to be evaluated (Fig. 6B). If the submitted file does not conform to the correct format, the web server notifies the user with the message "Failed to upload files" and requests the user to resubmit the compound in its correct format. After clicking the ***Submit*** button, the page redirects to a new page from which the user can download the result file (Fig. 6C).

## Conclusion

In this study, we have presented a novel tool called DeepSA for synthetic accessibility assessment of organic compounds, which offers advantages over previously known techniques. Since DeepSA is a deep learning model developed based on chemical language models, it reflects to some extent that the method of using SMILES to represent and extract features of compounds is not necessarily inferior to the graph representation method.

Although the model is considered successful, there is still room for improvement. Being lack of the data, DeepSA can’t learn real chemical reactions, the actual synthetic pathways, and their relative complexity, but only evaluates how complicated of those synthesis processes. DeepSA outperforms datasets that currently use the number of steps of retrosynthetic analysis as the standard, but has lower predictive power for molecules collected from the literature that have been evaluated by chemists. The reason that limits the generalizability of DeepSA is that the labels for datasets currently used come from the retrosynthetic analysis software evaluations and not all have been assessed by chemists. Determining the standard for evaluating the synthetic difficulty of a compound is still a matter of consideration. In this study, using 10 synthesis steps as the threshold for HS and ES is rather arbitrary, and the number of synthesis steps for a compound is easily influenced by the parameter settings of the retrosynthesis algorithm, especially the raw materials in the database.

Skoraczyński et al. evaluated the performance of the other compound synthesis accessibility prediction tools mentioned in this study, except for DeepSA and GASA [22], and the evaluation results were close to the test results obtained our work (Fig. 2). However, there is still room for improvement of this evaluation method to evaluate more reasonably.

To some extent, when evaluating the ease of synthesis of a compound, one should not only consider the number of steps in the synthesis reaction, but also focus on the yield of the products obtained from each step reaction, the cost of chemical reaction of each step with the experimental conditions, and so on. It is therefore imperative to create a completely new, clean, and informative dataset, but such a database is not currently available to us, which is a formidable challenge. We would like to fully account for the structural information of compounds with the variety of conditions that affect the synthetic reaction, but it clearly requires a great deal of time and patience to solve this problem. However, we believe that assessing the difficulty of synthesizing compounds in the current context of rapid advances in deep generation models is of very high academic and commercial value that merits our continued efforts to do deep thinking and research.

### Scientific contribution

In this study, we have developed a chemical language model called DeepSA for compound synthesis accessibility assessment. DeepSA has a high early enrichment rate in discriminating hard-to-synthesize molecules and could help users select less expensive molecules for synthesis. Meanwhile, DeepSA can be used as a useful filter for molecular generation models in computational chemistry.

### Supplementary Information

**Additional file 1: Table S1.** Detailed information of the parameter settings used for Retro*. **Table S2.** Detailed information of the data sets used in DeepSA. **Table S3.** Detailed information of the parameter settings used for DeepSA. **Table S4.** Performance comparison of the different DeepSA models on the external three test sets. **Table S5.** Generated three different randomized SMILES representations for each compound and extracted a total of 54 embeddings of the 18 compounds.

**Additional file 2: Fig. S1.** Embeddings of 18 different selected compounds in DeepSA.

## Data Availability

All the data sets and source code are publicly available through the GitHub (https://github.com/Shihang-Wang-58/DeepSA).
