# Supplementary material for: DeepSA: a deep-learning driven predictor of compound synthesis accessibility
Source: J Cheminform. 2023 Nov 2;15:103. doi: 10.1186/s13321-023-00771-3 (PMC10621138; doi:10.1186/s13321-023-00771-3)
Supplement: Supplementary file 1 — Additional file 1: Table S1. Detailed information of the parameter settings used for Retro*. Table S2. Detailed information of the data sets used in DeepSA. Table S3. Detailed information of the parameter settings used for DeepSA. Table S4. Performance comparison of the different DeepSA models on the external three test sets. Table S5. Generated three different randomized SMILES representations for each compound and extracted a total of 54 embeddings of the 18 compounds. [file 13321_2023_771_MOESM1_ESM.docx]

**DeepSA: A Deep-learning Driven Predictor of Compound Synthesis Accessibility**

Shihang Wang ^a,1^, Lin Wang ^a,1^, Fenglei Li ^b^, and Fang Bai ^a,b,c,*^

^a^ Shanghai Institute for Advanced Immunochemical Studies and School of Life Science and Technology, ShanghaiTech University, 393 Middle Huaxia Road, Shanghai, China, 201210

^b^ School of Information Science and Technology, ShanghaiTech University, 393 Middle Huaxia Road, Shanghai, China, 201210

^c^ Shanghai Clinical Research and Trial Center, Shanghai, China, 201210

^1^ These authors contributed equally

^*^ To whom correspondence should be addressed to

Fang Bai: [baifang@shanghaitech.edu.cn](mailto:baifang@shanghaitech.edu.cn)


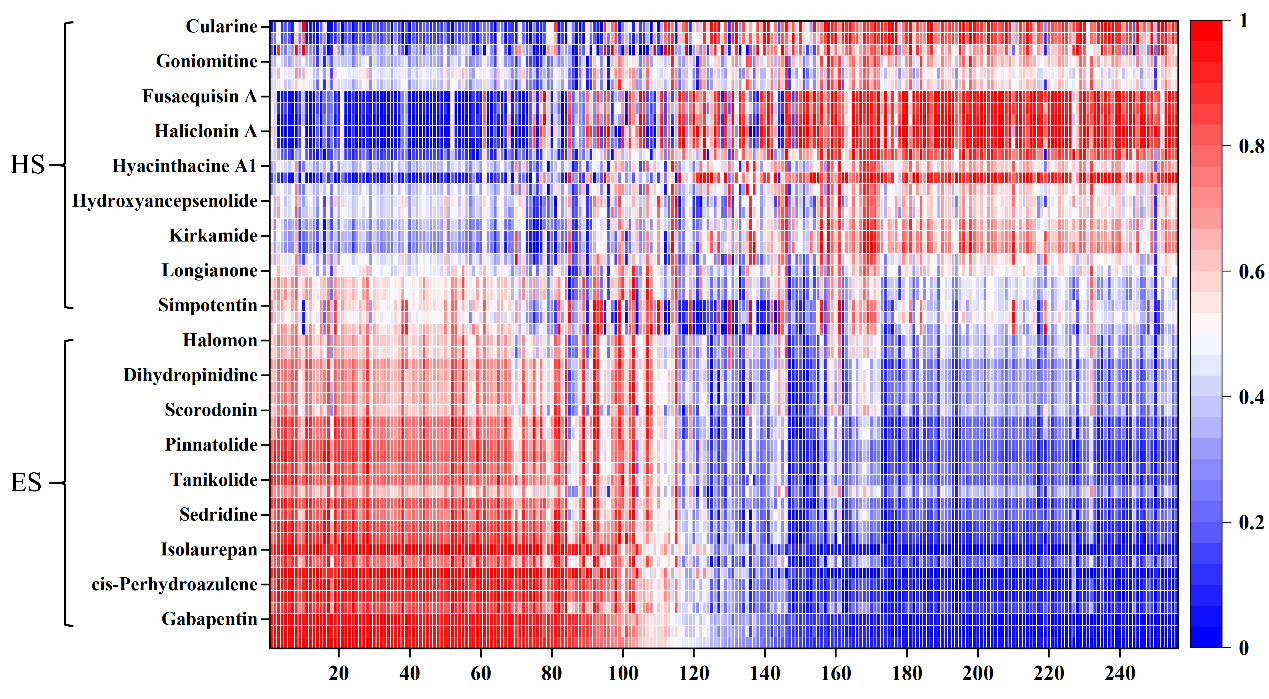


**Fig. S1** Embeddings of 18 different selected compounds in DeepSA. Three different randomized SMILES were generated for each molecule and represented as a matrix of 256 dimensional vectors. The matrix is normalized by column and uniformly ordered.

**Table S1** Detailed information of the parameter settings used for Retro*.

| **Hyperparameters** | **Vlues** |
| --- | --- |
| expansion_topk | 50 |
| iterations | 150 |
| starting_molecules | origin_dict.csv |
| mlp_templates | template_rules_1.dat |
| mlp_model_dump | saved_rollout_state_1_2048.ckpt |
| value_model | best_epoch_final_4.pt |
| fp_dim | 2048 |
| viz | False |

**Table S2** Detailed information of the data sets used in DeepSA.

| **Datasets** | **No. of compounds** | **No. of ES compounds** | **No. of HS compounds** |
| --- | --- | --- | --- |
| training set | 720,000 | 360,000 | 360,000 |
| test set | 80,000 | 40,000 | 40,000 |
| TS1 | 7,162 | 3,581 | 3,581 |
| TS2 | 30,348 | 17,348 | 13,000 |
| TS3 | 1,800 | 900 | 900 |

**Table S3** Detailed information of the parameter settings used for DeepSA.

| **Hyperparameters** | **Vlues** |
| --- | --- |
| embedding_size | 128 |
| max_position_embeddings | 512 |
| position_embedding_type | absolute |
| num_attention_heads | 4 |
| num_hidden_layers | 12 |
| attention_probs_dropout_prob | 0.1 |
| layer_norm_eps | 1e-12 |
| hidden_act | Gelu |
| hidden_dropout_prob | 0.1 |
| hidden_size | 256 |
| summary_activation | gelu |
| summary_last_dropout | 0.1 |
| summary_type | first |
| optim_type | adamw |
| learning_rate | 0.001 |
| weight_decay | 0.001 |
| loss_function | cross-entropy |
| batch_size | 256 |
| eval_batch_size_ratio | 4 |

**Table S4** Performance comparison of the different DeepSA models on the external three test sets.

| **Datasets** | **Model** | ***ACC*** | ***Recall*** | ***Precision*** | ***F–score*** | **AUROC** | **Threshold** |
| --- | --- | --- | --- | --- | --- | --- | --- |
| TS1 | DeepSA_ChemMTR | 0.995 | 0.998 | 0.991 | 0.995 | 1.000 | 0.50 |
|  | DeepSA_ChemMLM | 0.990 | 0.987 | 0.994 | 0.990 | 0.999 | 0.50 |
|  | DeepSA_MinBert | 0.995 | 1.000 | 0.989 | 0.995 | 1.000 | 0.50 |
|  | DeepSA_TinBert | 0.991 | 0.994 | 0.989 | 0.991 | 0.999 | 0.50 |
|  | DeepSA_RoBERTa | 0.995 | 1.000 | 0.989 | 0.995 | 1.000 | 0.50 |
|  | DeepSA_DeBERTa | 0.984 | 1.000 | 0.969 | 0.984 | 0.995 | 0.50 |
|  | DeepSA_GraphCodeBert | 0.994 | 0.999 | 0.988 | 0.994 | 1.000 | 0.50 |
|  | DeepSA_SmELECTRA | 0.995 | 1.000 | 0.990 | 0.995 | 1.000 | 0.50 |
| TS2 | DeepSA_ChemMTR | 0.838 | 0.750 | 0.855 | 0.799 | 0.910 | 0.50 |
|  | DeepSA_ChemMLM | 0.847 | 0.748 | 0.876 | 0.807 | 0.920 | 0.50 |
|  | DeepSA_MinBert | 0.831 | 0.736 | 0.849 | 0.789 | 0.902 | 0.50 |
|  | DeepSA_TinBert | 0.821 | 0.761 | 0.810 | 0.785 | 0.897 | 0.50 |
|  | DeepSA_RoBERTa | 0.840 | 0.745 | 0.862 | 0.799 | 0.910 | 0.50 |
|  | DeepSA_DeBERTa | 0.768 | 0.689 | 0.749 | 0.718 | 0.838 | 0.50 |
|  | DeepSA_GraphCodeBert | 0.830 | 0.721 | 0.861 | 0.785 | 0.902 | 0.50 |
|  | DeepSA_SmELECTRA | 0.838 | 0.730 | 0.871 | 0.795 | 0.913 | 0.50 |
| TS3 | DeepSA_ChemMTR | 0.789 | 0.719 | 0.837 | 0.773 | 0.880 | 0.50 |
|  | DeepSA_ChemMLM | 0.796 | 0.708 | 0.858 | 0.776 | 0.893 | 0.50 |
|  | DeepSA_MinBert | 0.802 | 0.748 | 0.838 | 0.790 | 0.882 | 0.50 |
|  | DeepSA_TinBert | 0.788 | 0.756 | 0.808 | 0.781 | 0.871 | 0.50 |
|  | DeepSA_RoBERTa | 0.816 | 0.769 | 0.849 | 0.807 | 0.891 | 0.50 |
|  | DeepSA_DeBERTa | 0.780 | 0.742 | 0.803 | 0.771 | 0.839 | 0.50 |
|  | DeepSA_GraphCodeBert | 0.807 | 0.748 | 0.849 | 0.795 | 0.886 | 0.50 |
|  | DeepSA_SmELECTRA | 0.817 | 0.761 | 0.861 | 0.808 | 0.896 | 0.50 |

**Table S5** Generated three different randomized SMILES representations for each compound and extracted a total of 54 embeddings of the 18 compounds.

| **ID** | **SMILES** |
| --- | --- |
| Gabapentin | C(CC1(CN)CCCCC1)(=O)O |
| Gabapentin | C(N)C1(CC(O)=O)CCCCC1 |
| Gabapentin | C(C(O)=O)C1(CCCCC1)CN |
| cis-Perhydroazulene | C1CCCC[C@H]2CCC[C@@H]12 |
| cis-Perhydroazulene | C1CC[C@@H]2[C@H]1CCCCC2 |
| cis-Perhydroazulene | C1[C@H]2[C@H](CCCCC2)CC1 |
| Isolaurepan | C1C[C@@H](CCC)O[C@@H](CCCCCC)CC1 |
| Isolaurepan | C(CCC)CC[C@@H]1O[C@@H](CCCC1)CCC |
| Isolaurepan | C1C[C@H](O[C@H](CC1)CCCCCC)CCC |
| Sedridine | O[C@H](C[C@H]1NCCCC1)C |
| Sedridine | [C@H]1(CCCCN1)C[C@H](C)O |
| Sedridine | C1C[C@@H](C[C@@H](O)C)NCC1 |
| Tanikolide | CCCCCCCCCCC[C@]1(OC(CCC1)=O)CO |
| Tanikolide | C(CC)CCCCCCCC[C@]1(CO)CCCC(=O)O1 |
| Tanikolide | O1C(=O)CCC[C@@]1(CO)CCCCCCCCCCC |
| Pinnatolide | C(=CC(CC1(C)OC(=O)CC1)=O)(C)C |
| Pinnatolide | O=C(C=C(C)C)CC1(CCC(O1)=O)C |
| Pinnatolide | O=C(C=C(C)C)CC1(C)CCC(O1)=O |
| Scorodonin | C(Cl)C#CC=C=CCO |
| Scorodonin | C(CCl)#CC=C=CCO |
| Scorodonin | C(CO)=C=CC#CCCl |
| Dihydropinidine | C1C(CCC)NC(CC1)C |
| Dihydropinidine | C(C1NC(CCC1)C)CC |
| Dihydropinidine | C(C)CC1CCCC(C)N1 |
| Halomon | CC([C@@H](CC[C@](C(=C)Cl)(CBr)Cl)Br)(C)Cl |
| Halomon | C(Cl)(C)([C@@H](CC[C@](Cl)(C(Cl)=C)CBr)Br)C |
| Halomon | ClC(C)(C)[C@H](Br)CC[C@@](Cl)(CBr)C(Cl)=C |
| Simpotentin | O[C@H]1[C@H]([C@@H]([C@@H](CO)O[C@H]1OC(CC(=O)O)CC(CCCCC)OC(CC(CC(O)CCC)O)=O)O)O |
| Simpotentin | O(C(=O)CC(CC(O)CCC)O)C(CC(CC(O)=O)O[C@H]1[C@@H](O)[C@@H](O)[C@@H]([C@@H](CO)O1)O)CCCCC |
| Simpotentin | C(CCCC)C(OC(=O)CC(CC(O)CCC)O)CC(O[C@@H]1O[C@@H]([C@@H](O)[C@H](O)[C@@H]1O)CO)CC(=O)O |
| Longianone | O=C1C=COC21CC(OC2)=O |
| Longianone | C1=CC(=O)C2(O1)CC(=O)OC2 |
| Longianone | C1(=O)CC2(CO1)C(C=CO2)=O |
| Kirkamide | O=C(C)N[C@H]1[C@@H](O)C=C(CO)[C@@H](O)[C@@H]1O |
| Kirkamide | O=C(C)N[C@@H]1[C@H]([C@@H](C(CO)=C[C@@H]1O)O)O |
| Kirkamide | C1=C(CO)[C@H]([C@@H]([C@H]([C@H]1O)NC(=O)C)O)O |
| Hydroxyancepsenolide | C(C[C@H]1[C@H]([C@H](C)OC1=O)O)CCCCCCCCCCC1=C[C@@H](OC1=O)C |
| Hydroxyancepsenolide | [C@H]1(C)C=C(C(=O)O1)CCCCCCCCCCCC[C@@H]1C(=O)O[C@H]([C@@H]1O)C |
| Hydroxyancepsenolide | O1[C@H](C=C(CCCCCCCCCCCC[C@@H]2C(=O)O[C@@H](C)[C@@H]2O)C1=O)C |
| Hyacinthacine A1 | C([C@H]1N2[C@H](CCC2)[C@H](O)[C@@H]1O)O |
| Hyacinthacine A1 | C1C[C@@H]2[C@@H]([C@H](O)[C@H](N2C1)CO)O |
| Hyacinthacine A1 | O[C@H]1[C@H]2CCCN2[C@H](CO)[C@H]1O |
| Haliclonin A | C1CCCCCCN(C=O)CC[C@@H]2[C@@H]3C[C@]4(CCC1)/C(C2=O)=C\[C@H](O)C/C=C\C/C=C/CCCN(C4)C3=O |
| Haliclonin A | [C@H]1(O)/C=C2\[C@@]34C[C@@H]([C@H](C2=O)CCN(CCCCCCCCCC4)C=O)C(N(CCC/C=C/C/C=C\C1)C3)=O |
| Haliclonin A | C1N2C[C@@]34C[C@@H]([C@@H](CCN(CCCCCCCCCC3)C=O)C(/C4=C/[C@H](O)C/C=C\C/C=C\CC1)=O)C2=O |
| Fusaequisin A | [C@H]1([C@H]([C@H](/C=C(C)/C=C/C(C(C)C(C)=O)OC)[C@@H](OC)O1)C)/C(C)=C/C=C/C(CCCC)C |
| Fusaequisin A | C(CCC)C(C)/C=C/C=C(/[C@H]1O[C@H](OC)[C@@H](/C=C(/C=C/C(C(C(=O)C)C)OC)C)[C@@H]1C)C |
| Fusaequisin A | [C@@H]1([C@@H]([C@@H](/C(C)=C/C=C/C(CCCC)C)O[C@@H]1OC)C)/C=C(C)/C=C/C(C(C)C(C)=O)OC |
| Goniomitine | [C@@]12(CC)[C@H](n3c(c(CCO)c4ccccc43)CC2)NCCC1 |
| Goniomitine | C(O)Cc1c2n([C@@H]3NCCC[C@]3(CC)CC2)c2ccccc12 |
| Goniomitine | c1cccc2c(CCO)c3CC[C@]4(CCCN[C@H]4n3c12)CC |
| Cularine | c12c(OC)ccc3CCN([C@@H](Cc4c(cc(c(c4)OC)OC)O2)c13)C |
| Cularine | c1c2c(cc(OC)c1OC)C[C@@H]1N(CCc3c1c(O2)c(OC)cc3)C |
| Cularine | c1c2c(Oc3c4c(CCN([C@H]4C2)C)ccc3OC)cc(OC)c1OC |
