# Supplementary figures and images for: DeepSA: a deep-learning driven predictor of compound synthesis accessibility
Source: J Cheminform. 2023 Nov 2;15:103. doi: 10.1186/s13321-023-00771-3 (PMC10621138; doi:10.1186/s13321-023-00771-3)

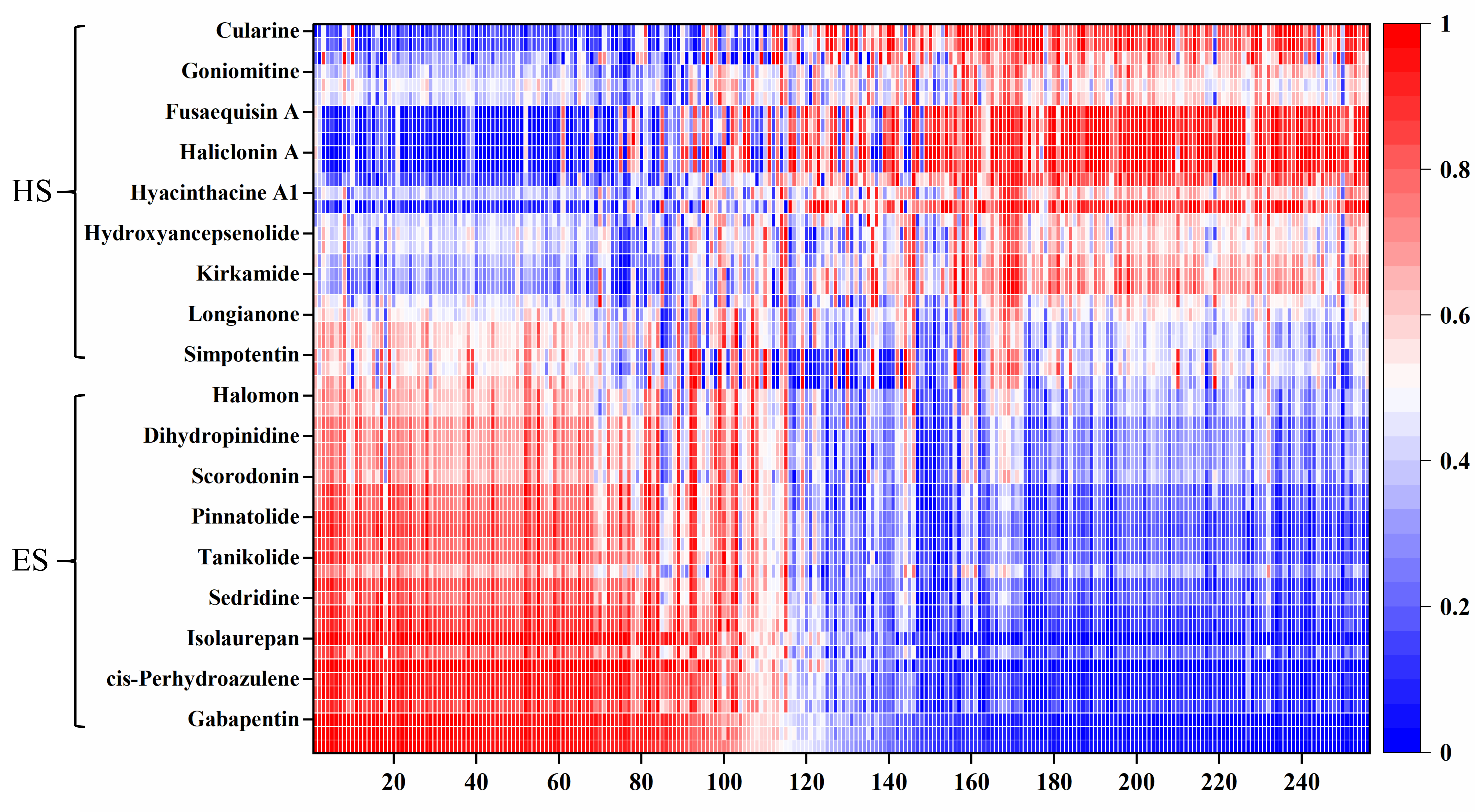

Supplement: Supplementary file 2 — Additional file 2: Fig. S1. Embeddings of 18 different selected compounds in DeepSA. [file 13321_2023_771_MOESM2_ESM.tif]
